# Molecular Bases of Brain Preconditioning

**DOI:** 10.3389/fnins.2017.00427

**Published:** 2017-07-25

**Authors:** Oleg G. Deryagin, Svetlana A. Gavrilova, Khalil L. Gainutdinov, Anna V. Golubeva, Vyatcheslav V. Andrianov, Guzel G. Yafarova, Sergey V. Buravkov, Vladimir B. Koshelev

**Affiliations:** ^1^Department of Physiology and General Pathology, Medical Faculty, Lomonosov Moscow State University Moscow, Russia; ^2^Laboratory of Neurorehabilitation of Motor Disorders, Institute of Fundamental Medicine and Biology, Kazan Federal University Kazan, Russia; ^3^Laboratory of Spin Physics and Spin Chemistry, Zavoisky Physical-Technical Institute of the Russian Academy of Sciences Kazan, Russia; ^4^Research Laboratory of Cellular Structure and Tissue Imaging Analysis, Medical Faculty, Lomonosov Moscow State University Moscow, Russia

**Keywords:** ischemic preconditioning, ATP-sensitive potassium channels, nitric oxide, mitochondria, neuroprotection

## Abstract

Preconditioning of the brain induces tolerance to the damaging effects of ischemia and prevents cell death in ischemic penumbra. The development of this phenomenon is mediated by mitochondrial adenosine triphosphate-sensitive potassium (${\text{K}}_{\text{ATP}}^{+}$) channels and nitric oxide signaling (NO). The aim of this study was to investigate the dynamics of molecular changes in mitochondria after ischemic preconditioning (IP) and the effect of pharmacological preconditioning (PhP) with the ${\text{K}}_{\text{ATP}}^{+}$-channels opener diazoxide on NO levels after ischemic stroke in rats. Immunofluorescence-histochemistry and laser-confocal microscopy were applied to evaluate the cortical expression of electron transport chain enzymes, mitochondrial ${\text{K}}_{\text{ATP}}^{+}$-channels, neuronal and inducible NO-synthases, as well as the dynamics of nitrosylation and nitration of proteins in rats during the early and delayed phases of IP. NO cerebral content was studied with electron paramagnetic resonance (EPR) spectroscopy using spin trapping. We found that 24 h after IP in rats, there is a two-fold decrease in expression of mitochondrial ${\text{K}}_{\text{ATP}}^{+}$-channels (*p* = 0.012) in nervous tissue, a comparable increase in expression of cytochrome c oxidase (*p* = 0.008), and a decrease in intensity of protein S-nitrosylation and nitration (*p* = 0.0004 and *p* = 0.001, respectively). PhP led to a 56% reduction of free NO concentration 72 h after ischemic stroke simulation (*p* = 0.002). We attribute this result to the restructuring of tissue energy metabolism, namely the provision of increased catalytic sites to mitochondria and the increased elimination of NO, which prevents a decrease in cell sensitivity to oxygen during subsequent periods of severe ischemia.

## Introduction

The resistance of the brain to the interruption of its blood supply and subsequent hypoxia can be increased by a pre-exposure to short episodes of ischemia/reperfusion or hypoxia (Bolanos and Almeida, 1999; Manukhina et al., 1999; Shmonin et al., 2012; Rybnikova and Samoilov, 2015), short periods of hypothermia (Maslov et al., 2012) and other moderate stress effects which are capable of activating endogenous protective mechanisms and increasing resistance to subsequent severe ischemia (Samoilenkova et al., 2008; Ding et al., 2012; Lim and Hausenloy, 2012). This phenomenon is called “preconditioning.” There is another phenomenon—post-conditioning, in which the protective impulse, e.g., brief interruptions of reperfusion, is applied after the onset of ischemia (Rybnikova and Samoilov, 2015; Wang et al., 2015).

Different cellular mechanisms are involved in realization of the protective effects of preconditioning and post-conditioning. Post-conditioning is mediated by functional responses of the Na^+^/Ca^2+^ exchangers, the plasma membrane Ca^2+^-ATPase, the Na^+^/H^+^ exchange, the Na^+^/K^+^/2Cl^−^ co-transport and the acid-sensing cation channels, which main function is normalization of intracellular pH and calcium levels, and a number of signaling cascades (Cuomo et al., 2015).

The primary mechanism of the preconditioning-induced neuroprotection is ${\text{K}}_{\text{ATP}}^{+}$-channels opening (Samoilenkova et al., 2008). A decrease in ATP levels during ischemia promotes the activation of ${\text{K}}_{\text{ATP}}^{+}$-channels in the cell membrane, which restores the low concentrations of Na^+^ and Ca^2+^ in the cytosol and restrains excessive depolarization. Kir6.2 is considered to be a predominant pore-forming subunit of neuronal plasmalemmal ${\text{K}}_{\text{ATP}}^{+}$-channel (Yamada et al., 2001). The opening of ${\text{K}}_{\text{ATP}}^{+}$-channels in the mitochondrial inner membrane is associated with the prevention of mitochondrial calcium overload (Murata et al., 2001) and the subsequent preservation of mitochondrial function (Mironova et al., 2007; Correia et al., 2010). Early immunohistochemical studies identified a mitochondrial subunit of this channel as Kir6.1 (Lacza et al., 2003b; Singh et al., 2003). However, further proteomic studies did not confirm these results (Brustovetsky et al., 2005), but identified the potential structural basis of mitochondrial ${\text{K}}_{\text{ATP}}^{+}$-channel as Kir1.1 (Foster et al., 2008, 2012; Foster and Coetzee, 2016), which has an N-terminal mitochondrial targeting signal and is encoded by *KCNJ1* (potassium voltage-gated channel, subfamily J, member 1) gene. In cardiomyocytes, Kir1.1 is localized only in the crista membranes, mostly in the center of mitochondria and the channel subunits are frequently grouped together (Talanov et al., 2016). Such clustering corresponds to the specific feature of a mitochondrial Kir allowing a 10-times higher sensitivity to ATP inhibition in mitochondria compared to liposomes and lipid bilayers (Mironova et al., 2004). Compared to liver or heart, brain mitochondria expresses six- to seven-times more ${\text{K}}_{\text{ATP}}^{+}$-channels, which opening is accompanied by a larger change in respiration (Bajgar et al., 2001). Although the expression of regulatory subunits SUR2A and SUR2B has been demonstrated in mitochondrial membranes (Zhou et al., 2007), Kir1.1 doesn't form a complex with SUR2B, but has the same sensitivity to glibenclamide as the native channel (Konstas et al., 2002). It was also hypothesized that mitochondrial ${\text{K}}_{\text{ATP}}^{+}$-channels may have a smaller molecular weight SUR variant (Lacza et al., 2003a).

Alternatively, it is suggested that a succinate dehydrogenase or resporatory complex II is the regulatory component of mitochondrial ${\text{K}}_{\text{ATP}}^{+}$-channels within a super-complex of proteins with an inverse relationship between complex II and mitochondrial ${\text{K}}_{\text{ATP}}^{+}$-channel activities (Wojtovich et al., 2013). Complex II has also been recognized as a modulator of a superoxide production by respiratory chain complexes I and III (Dröse, 2013). This fact is important for understanding of functional consequences of a reactive oxygen species (ROS) production, which might be cardioprotective when ROS formed in complex III, but deleterious in case of the ROS produced in complex I (Madungwe et al., 2016). Generation of ROS at low levels can mediate the protective effect of preconditioning (Kalogeris et al., 2014) apparently by the activation of ${\text{K}}_{\text{ATP}}^{+}$-channels. ROS oxidize thiol groups of mitochondrial protein kinase Cε (PKCε; Korichneva et al., 2002), which is co-localized with ${\text{K}}_{\text{ATP}}^{+}$-channels in the inner mitochondrial membrane (Jabůrek et al., 2006). Activated PKCε phosphorylates ${\text{K}}_{\text{ATP}}^{+}$-channels leading them to consistently open state (Garlid et al., 2013). An increase of PKCε interaction with cytochrome c oxidase subunit IV has also been observed under conditions of myocardial IP and is associated with enhanced respiratory complex IV activity (Guo et al., 2007). However, tissue oxygen consumption can be inhibited due to NO overproduction and binding to cytochrome c oxidase (Brown and Cooper, 1994). The properties of NO action depend on the intensity of its production and the physiological state of the surrounding tissue. NO overproduction in stroke causes damage to structural and regulatory components of cells (Bolanos and Almeida, 1999; Jung et al., 2006; Terpolilli et al., 2012). Moderate activation of NO during preconditioning may exert a neuroprotective effect (Schulz and Ferdinandy, 2013), activating antioxidant enzymes, triggering antiapoptotic mechanisms, and increasing cerebral blood flow (Jung et al., 2006; Terpolilli et al., 2012). The protective effect of moderate NO production may also be mediated by an interaction with ${\text{K}}_{\text{ATP}}^{+}$-channels opening (Sasaki et al., 2000). An inverse relation between these elements in the context of the neuroprotective effect of preconditioning has not been yet proven because of the absence of experimental approaches, precise methods of NO detection and verification of results obtained *in vivo*.

Different pre- and post-conditioning strategies has been tested in clinical settings since 1990s (Calabrese, 2016). However, the clinical studies have shown controversial results (Thuret et al., 2014). The protective effect of preconditioning is known to be lost in old age and in metabolic disorders (Rana et al., 2015; Calabrese, 2016), but it is possible to restore it by physical exercises and caloric restriction. There is a need to define the therapeutic targets for rapid pharmacological restoration of the responsiveness to preconditioning and it requires an understanding of key molecular events starting from the point of the protective changes induction, through the development of protective phenotype to the final realization of protective mechanisms under ischemia conditions. Thus, we conducted the present study to investigate the mitochondrial protein composition in brain cortex of rats after IP and to evaluate the changes of NO production in two models of ischemia—mild ischemia with reperfusion (IP) and the severe ischemia without reperfusion (ischemic stroke). Preliminary ${\text{K}}_{\text{ATP}}^{+}$-channel blockade and opening were used to study the relationship between ${\text{K}}_{\text{ATP}}^{+}$-channels and the NO system.

## Materials and methods

### Animals

Experiments were performed with 5 months old naïve male albino rats (Rattus norvegicus) weighing 300–500 g (*n* = 102) and kept under standard conditions (light regimen of 12/12 h, day/night). All manipulations were performed under general anesthesia with a long-acting aliphatic hypnotic drug chloral hydrate [ChlH, 400 mg/kg intraperitoneally (i/p)]. The study was conducted in accordance with and approved by the Bioethics Committee of Lomonosov Moscow State University and in accordance with the ARRIVE guidelines.

### Study of the IP molecular basis

**Figure 1:**
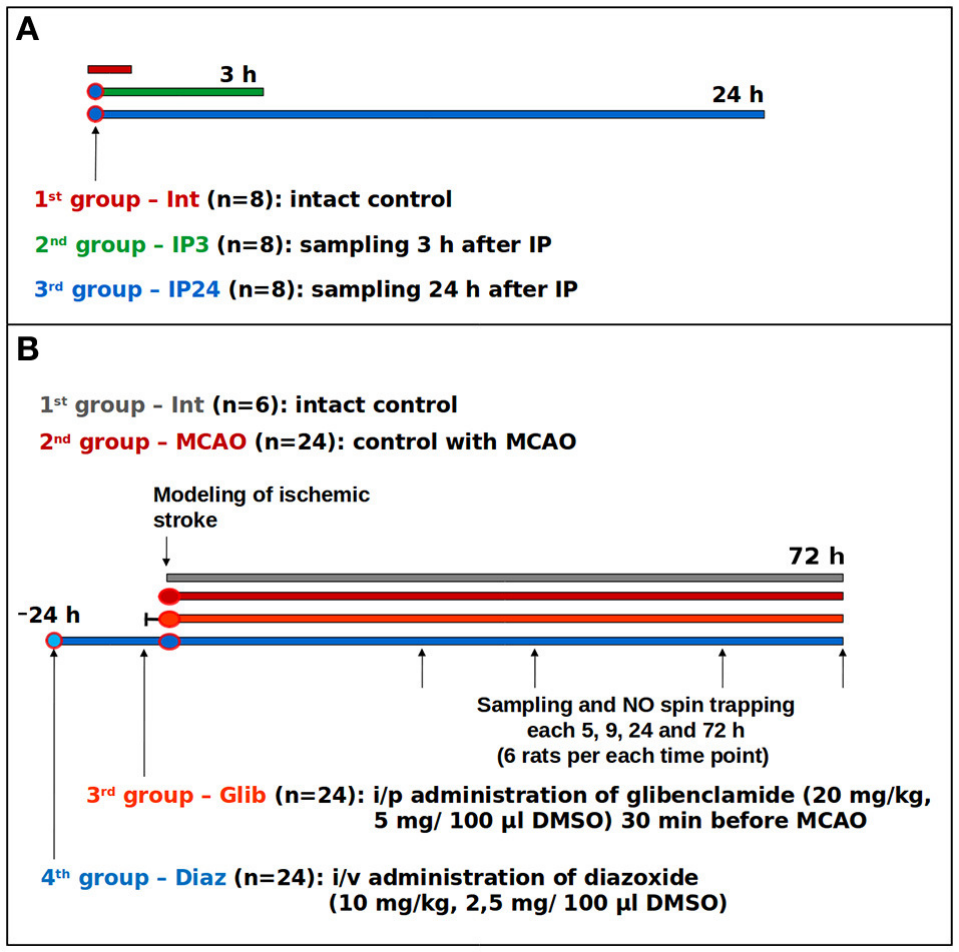
Study protocols: **(A)** study on the effects of early and delayed IP phases on the expression of immunohistochemical markers; **(B)** study of glibenclamide and diazoxide effects on NO levels in the brain of the rats with ischemic stroke.

In the first experiment, we studied the effects of IP on the cortical expression of electron transport chain enzymes, mitochondrial ${\text{K}}_{\text{ATP}}^{+}$-channels, neuronal, and inducible NO-synthases, as well as the dynamics of nitrosylation and nitration of proteins during early and delayed phases of IP (Figure 1A). IP was performed by alternate ligation of the right and left common carotid arteries for 5 min followed by 5 min of reperfusion. Overall 6 cycles were repeated for each animal within 1 h. The early (3 h) and the delayed (24 h) phases of the protective IP effect were subsequently studied. An indirect immunohistochemical method was applied with secondary antibodies (Table 1) labeled with fluorochromes—fluorescein (FITC) and phycoerythrin (PE). Karnua solution was used as fixative. For the S-nitrosoCys study brain tissue samples were fixed in a solution of 4% paraformaldehyde and 1% of glutaraldehyde. Alcohol-chloroform processing and paraffin-embedding were carried out before slicing paraffin blocks into sections of 3 μm thickness. After deparaffinization, antigen retrieval was performed in microwave. Permeabilization was performed sequentially with 0.3, 0.2, and 0.1% solutions of TritonX-100 in sodium phosphate buffer (PBS-T). Slices were incubated with 5% goat serum in PBS-T 0.3% for 30 min at room temperature. Then slices were rinsed 3 times with PBS, permeabilization was repeated and they were incubated at 37°C for 1 h with a solution of primary antibodies in Antibody Diluent (ab64211—Abcam plc, Cambridge, UK). For double staining, repeated washing and incubation with the next primary antibody solution were performed. After washing of primary antibodies, slices were incubated at room temperature with secondary antibodies dissolved in deionized water. After secondary antibody washing, coverslips were mounted over the slides using UltraCruz Hard-set mounting medium (sc-359850—Santa Cruz Biotechnology, Santa Cruz, CA, USA).

**Table 1:** Panel of antibodies.

**Marker**	**Primary antibody**	**Secondary antibody**
COX1	Mouse monoclonal [1D6E1A8] anti-MTCO1 antibody, mitochondrial marker (ab14705^*^), 1:200	Goat anti-mouse IgG_2a_-FITC (sc-2079^**^), 1:100
SDHA	Rabbit polyclonal anti-SDHA antibody (sc-98253), 1:50	Goat anti-rabbit IgG-PE (sc-3739), 1:100
KCNJ1	Rabbit polyclonal anti-KCNJ1 antibody, cytoplasmic domain (ab80967), 1:20	Goat anti-rabbit IgG-PE (sc-3739), 1:100
nNOS	Rabbit polyclonal anti-nNOS antibody (ab106417), 1:200	Goat anti-rabbit IgG-PE (sc-3739), 1:100
iNOS	Mouse monoclonal [C-11] anti-NOS2 antibody (sc-7271), 1:50	Goat anti-mouse IgG_1_-FITC (sc-2078), 1:100
S-nitrosoCys	Mouse monoclonal [HY8E12] anti-S-nitrosocysteine (conjugated) antibody (ab94930), 1:1000	Goat anti-mouse IgG_1_-FITC (sc-2078), 1:100
3-nitroTyr	Mouse monoclonal [39B6] anti-3-Nitrotyrosine antibody (ab61392), 1:200	Goat anti-mouse IgG_2a_-FITC (sc-2079), 1:100

* *≪ab≫ product IDs refer to Abcam plc, Cambridge, UK*.

** *≪SC-≫ product IDs refer to Santa Cruz Biotechnology, Santa Cruz, CA, USA*.

Scanning of slices was performed using a laser scanning confocal Carl Zeiss LSM780 microscope, with 5 images taken lengthwise the frontal section of the frontal lobe cortex for each slide and with one slide stained with primary antibodies per each animal. A fixed scanning mode was used for evaluation of all the slides in order to minimize the variability of fluorescence intensities (normalization was not possible due to unstable DAPI staining pattern): Plan-Apochromat 63x/1.40 Oil DIC M27 lens; 40 micron pinhole; fluorescent signal filtering in 499–560 nm range. The following laser intensity parameters were used for fluorescence excitation: 355 nm—5%; 561 nm—2%; 488 nm—10%. The expression of each marker was studied at the same day with the interchange of slides from different experimental groups to prevent the influence of natural fluorescence extinction. The subsequent quantitative analysis of results was done in Image-Pro v.4.5 program. Fluorescence intensity was calculated as the percentage of specific fluorescence points in the area of interest. The snapshots of pyramidal neurons were transferred into 16-bit images and processed through a macro that enables deduction of a non-specific background fluorescence from the analysis results, using the brain slices stained only with secondary antibodies as a control. This approach was chosen because of the background auto-fluorescence in paraffin blocks, the maximum values of which were observed in glutaraldehyde fixation. A qualitative evaluation of fluorescence intensity using the 5-point scale was also applied in order to confirm the quantitative analysis. Both methods showed comparable results, with a slightly lower significance of differences measured in the qualitative evaluation (data not shown). Co-localization of markers was defined in ImageJ program with subsequent analysis of 0.19–2.0 μm^2^ areas.

### Study of the PhP effect on brain NO content

In the second experiment, we examined the effects of preliminary ${\text{K}}_{\text{ATP}}^{+}$-channels blockade and opening on free cerebral NO following middle cerebral artery (MCA) occlusion (MCAO; Figure 1B). The objective was to evaluate an integrated contribution of ${\text{K}}_{\text{ATP}}^{+}$-channels in regulation of NO levels. PhP was performed by injecting a solution of a non-selective opener of ${\text{K}}_{\text{ATP}}^{+}$-channels, diazoxide (Sigma-Aldrich Rus LLC, Moscow, Russia), at a dose of 10 mg/kg [2.5 mg/100 mcl of dimethyl sulfoxide (DMSO)] in the sublingual vein (i/v) 24 h prior to MCAO. For the purposes of non-selective blockade of ${\text{K}}_{\text{ATP}}^{+}$-channels, i/p administration of glibenclamide (Sigma-Aldrich Rus LLC, Moscow, Russia, 20 mg/kg, 5 mg/100 mcl DMSO) was applied 30 min before MCAO. The selection of dosage and injection time was based on literature data (Marshall et al., 1993; Liu et al., 2002; Shimizu et al., 2002).

Ischemic infarction in the fronto-parietal region of cerebral cortex was induced by electrocoagulation of the distal branch of left MCA and the adjacent vein, with simultaneous ligation of the ipsilateral carotid artery. This model of ischemic stroke was chosen because of its good reproducibility in experiments which demonstrated a 30–45% decrease of necrotic area after IP or PhP (Samoilenkova et al., 2007; Deryagin et al., 2016). The MCA coagulation altitude was chosen so that necrosis developed mainly in the frontoparietal cortex, without affecting subcortical structures, thereby avoiding complications of visceral functions and animal death. Simultaneous coagulation of the adjacent vein and ligation of the ipsilateral carotid artery stabilizes the size of the ischemic area, which also reduces the number of animals required for the experiment.

EPR spectroscopy with spin trapping (Mikoyan et al., 1997; Vanin et al., 2003) was used to measure NO cerebral content. For this purpose, animals received components of a trap 30 min before sampling: 500 mg/kg of sodium diethyldithiocarbamate (DETC) (Sigma-Aldrich Rus LLC, Moscow, Russia) in the amount of 8.4 ml/kg i/p, and a mixture of solutions of 37.5 mg/kg FeSO_4_, 187.5 mg/kg of sodium citrate in a total volume of 6.6 ml/kg subcutaneously. As, a result of their interaction within the body, DETC_2_-Fe^2+^ water-insoluble complex is formed, capable of capturing NO with the formation of (DETC)_2_-Fe^2+^-NO stable radical, which is detected by EPR [Bruker ER 200E SRC in X-band (9.50 GHz) spectrometer at 77 oC]. The method was described by us earlier (Gainutdinov et al., 2011, 2013). The trapping of NO was introduced 30 min before decapitation. For each time point (0, 5, 9, 24, and 72 h) in each group, six independent measurements were done. These time points were chosen, taking into account the activity of constitutive and inducible NO-synthases in the development of ischemic stroke: 5 and 9 h cover the period of constitutive NO-synthase activity, while the 24- and 72-h points make it possible to estimate the activity of inducible NO-synthase (Kitamura et al., 1998). The brain fragments were immediately frozen in separate plastic containers, overall 4 probes from each animal: the ischemic region of cortex in the left cerebral hemisphere; the remainder cortex of the left cerebral hemisphere; cortex of the right cerebral hemisphere; and cerebellum. The samples were weighted before the experiments and their average mass was 100 mg. Amplitude of EPR spectra was normalized on the mass of sample and on the EPR signal amplitude of standard sample. As a standard sample, we use the TEMPO radical solution (8.89 mM/l, 58^*^10^17^ spins/cm^3^, S = 1/2, g~2.0023; Barr et al., 2000).

### Statistical analysis

Statistical processing was performed using Excel 2010 and SPSS 17.0 software packages for Windows. Data distribution was evaluated with Shapiro–Wilk test and it didn't meet the assumption of normality. Thus, non-parametric statistical methods were applied. When comparing more than two independent samples, Kruskal–Wallis test (H-criterion) was used. When comparing two independent samples, Mann–Whitney test (U-criterion) was used. Data of graphs is presented as Median (within IQR for the box plot graph) with Min and Max whiskers. Differences were considered significant with the permissible error probability (two-tailed *p*-value) of <0.05.

## Results

### Effect of delayed phase of IP on expression of COX1, SDHA, KCNJ1, nNOS, and iNOS

**Figure 2:**
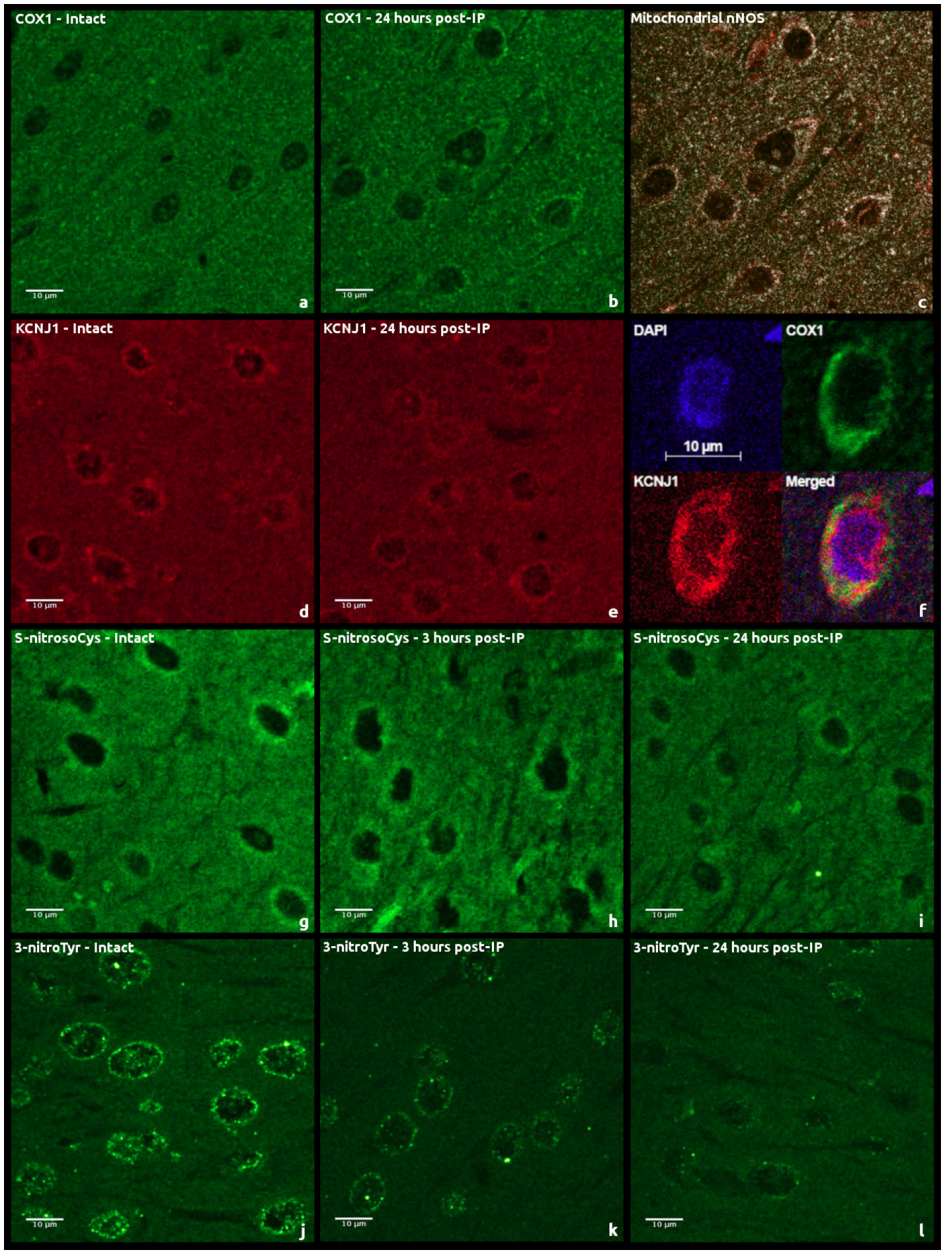
Examples of immunohistochemical staining of rat cerebral cortex sections using second antibodies labeled with fluorochromes: **(a,b)** anti-COX1 antibody staining; **(c)** COX1 and nNOS co-localization; **(d,e)** anti-KCNJ1 antibody staining; **(f)** KCNJ1 and COX1 co-localization; **(g–i)** anti-S-nitrosylation (S-nitrosoCys) antibody staining; **(j–l)**, anti-tyrosine nitrosylation (3-nitroTyr) antibody staining.

**Figure 3:**
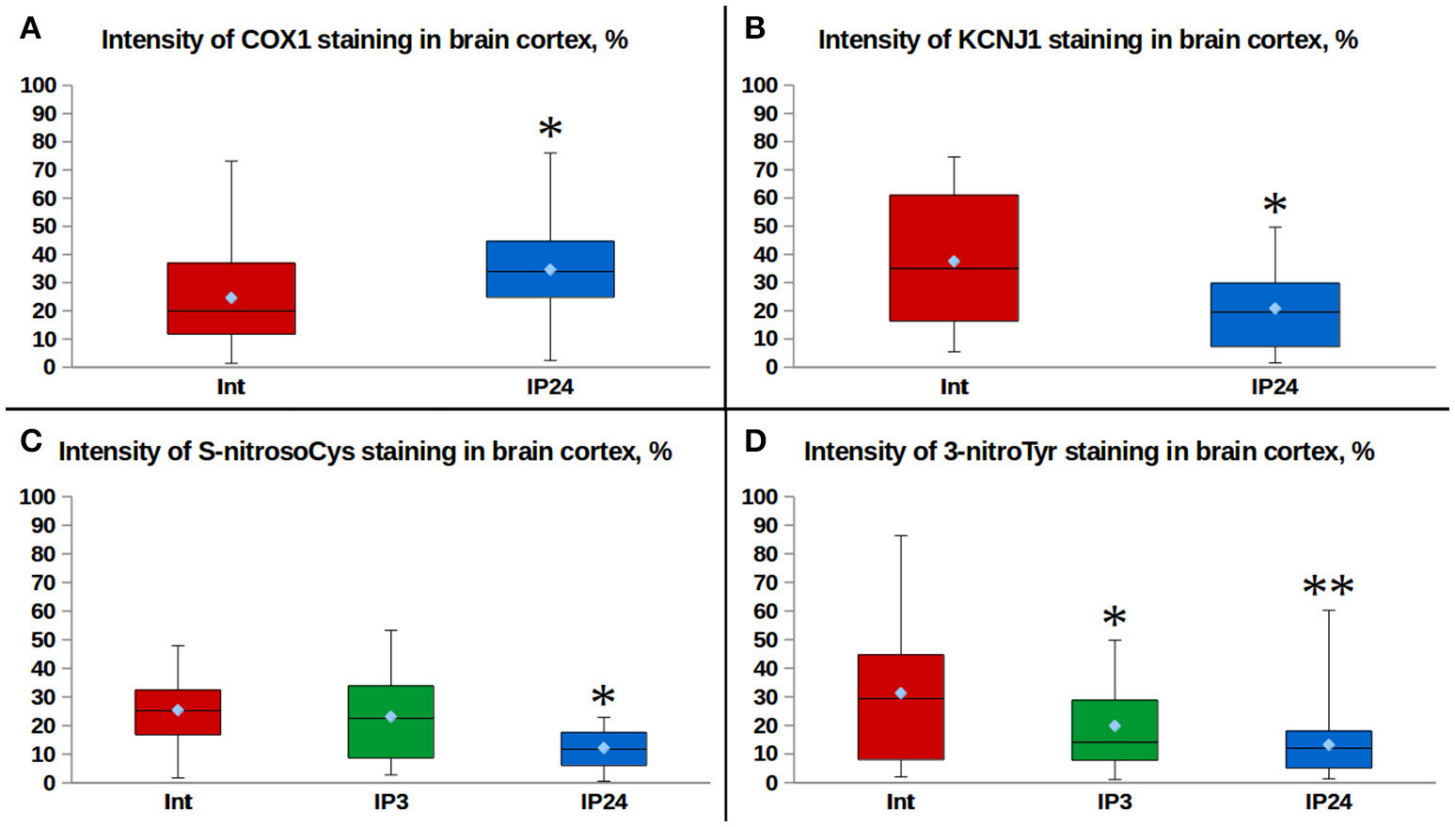
Comparison of fluorescence intensities: **(A)** effects of delayed IP phase on the expression of COX1 in rat cerebral cortex cells. ^*^p = 0.008, Mann–Whitney test (Int. vs. IP24); **(B)** effects of delayed IP phase on the expression of KCNJ1 in rat cerebral cortex cells. ^*^p = 0.012, Mann–Whitney test (Int. vs. IP24); **(C)** effects of delayed IP phase on cysteine S-nitrosylation levels in rat cerebral cortex cells. ^*^p = 0.0004, Mann–Whitney test (Int. vs. IP24); *p* = 0.001, Mann–Whitney test (IP3. vs. IP24); **(D)** effects of delayed IP phase on tyrosine nitration levels in rat cerebral cortex cells. ^*^p = 0.053, Mann–Whitney test (Int. vs. IP3); ^**^p = 0.001, Mann–Whitney test (Int. vs. IP24); *p* = 0.019, Mann–Whitney test (IP3. vs. IP24).

Moderately expressed cytoplasmic and predominantly perinuclear cytochrome c oxidase subunit 1 (COX1) expression of granular pattern was observed in cerebral cortical regions of intact animals (Figures 2a,b). A similar kind of a weak granular COX1 staining was detected in the neuropil. Twenty-four hours after performing IP, the median intensity of fluorescence was 70% higher than in the control group (Mann–Whitney test, *p* = 0.008, Figure 3A). However, a staining of succinate dehydrogenase flavoprotein subunit (SDHA) revealed no changes in intensity of SDHA expression in all the animal groups (*p* > 0.05, data not shown). An expression of mitochondrial ${\text{K}}_{\text{ATP}}^{+}$-channels (KCNJ1) was moderate and varied between animals (Figures 2d,e). KCNJ1 staining was co-localized with COX1 (Figure 2f). IP led to a decrease in median KCNJ1 fluorescence intensity by 44% in cerebral cortex in its delayed phase, as compared to the intact group (Mann–Whitney test, *p* = 0.012, Figure 3B).

Expression of neuronal NO-synthase (nNOS) had cytoplasmic staining pattern, mostly fine-grained. The intensity of fluorescence varied from moderate to strong, more intense in the cells located closer to arterioles. Expression of nNOS was comparable in intact and IP24 groups (Mann–Whitney test, *p* = 0.376). COX1/nNOS co-localization analysis made it possible to evaluate the expression level of mitochondrial nNOS fraction (Figure 2c). There was no statistically significant difference found between intact rats and IP24 group (Mann–Whitney test, *p* = 0.261). An evaluation of the area and the perimeter of double stained zones in mitochondria revealed no differences between the two groups. Both in intact rats and after IP, only minimal expression of inducible NO-synthase (iNOS) was detected in cerebral cortex, which does not allow estimating the staining results reliably.

### Effect of early and delayed phases of IP on processes of nitrosylation and nitration

A variable pattern of cysteine S-nitrosylation marker (S-nitrosoCys) fluorescence was observed in the cerebral cortex specimens. Its cytoplasmic staining was mostly diffuse, with the greatest intensity in intact animals and in rats 3 h after IP (Figures 2g–i). The quantitative analysis of data revealed a 2-fold (Figure 3C) statistically significant decrease in the median S-nitrosoCys fluorescence intensity in delayed phase of IP, in comparison with both the intact and the IP3 groups (Mann–Whitney test, *p* = 0.0004 and *p* = 0.001, respectively). When specimens were stained with tyrosine nitration marker (3-nitroTyr), a weak diffuse perinuclear fluorescence was observed, as well as the pronounced dot-like staining in both the perinuclear zone and in the nucleus (Figures 2j–l). The median intensity was higher in intact animals, 2- and 2.5-fold more prominent than at 3 and 24 h after IP, respectively (Mann–Whitney test, *p* = 0.053 and *p* = 0.001, Figure 3D).

### Impact of ${\text{K}}_{\text{ATP}}^{+}$-channels on NO cerebral content in rats with ischemic stroke

**Figure 4:**
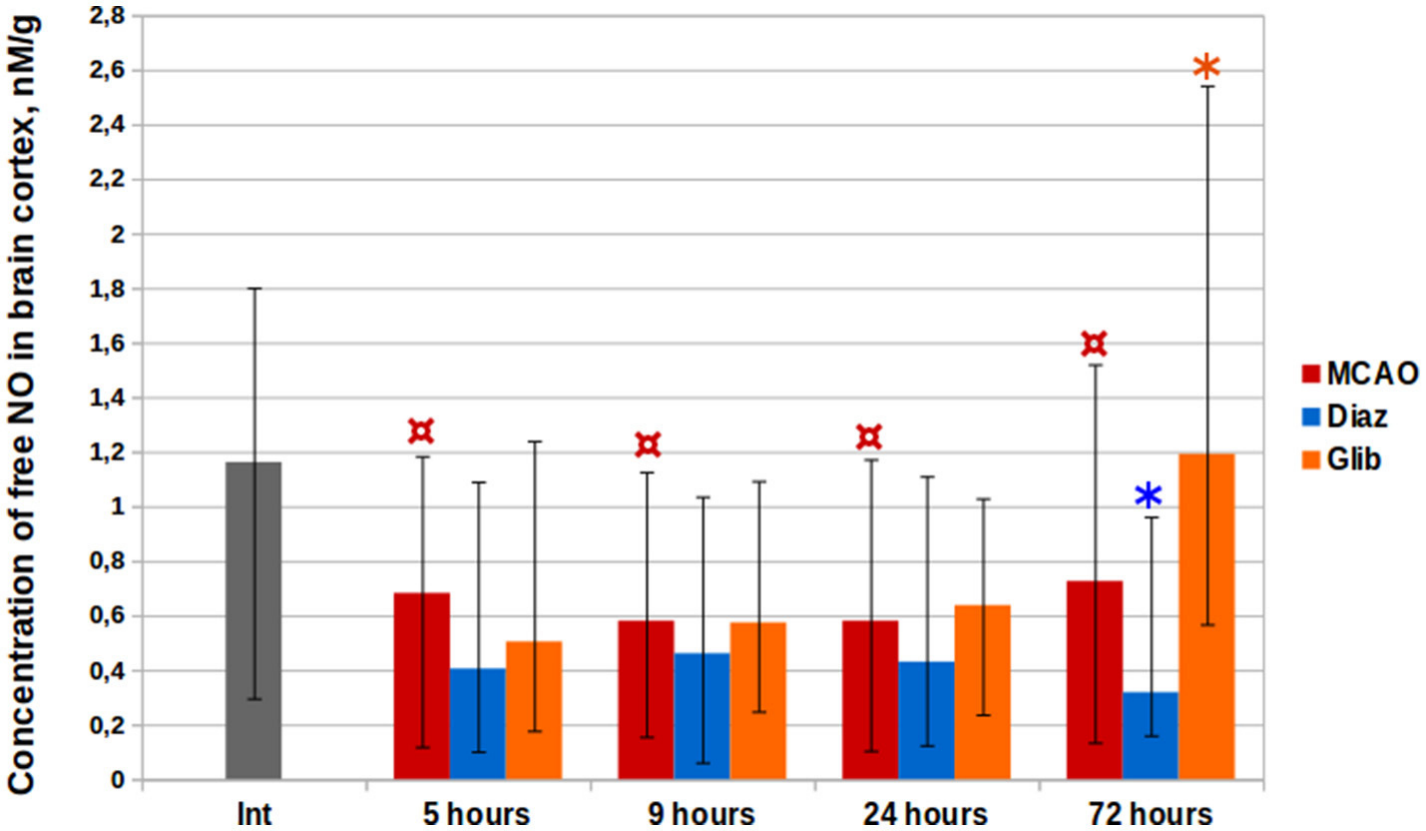
Complex (DETC)_2_-Fe^2+^-NO time history in cerebral cortical structures in the rats with ischemic stroke. ^*^p < 0.05, Mann-Whitney test (MCAO vs. Glib, 72 h; MCAO vs. Diaz, 72 h); ^¤^*p* < 0.0001, Kruskal–Wallis test (MCAO, int. vs. 5, 9, 24, and 72 h).

The cortical levels of (DETC)_2_-Fe^2+^-NO complex in control groups of rats with MCAO were two times less than in intact animals at all the time points (Kruskal–Wallis test, *p* < 0.0001, Figure 4). The median level of NO in the cerebral cortex of intact animals amounted to 1.16 nM/g (IQR = 0.95–1.52). Five hours after MCAO, the minimal median NO concentration of 0.23 nM/g (IQR = 0.17–0.27) was observed in the core of ischemia increasing with distance from it in penumbra (0.42 nM/g, IQR = 0.20–0.71) to 0.70 nM/g (IQR = 0.64–0.74) in the contralateral hemisphere and to 0.81 nM/g (IQR = 0.71–0.91) in the cerebellum. The median NO concentrations at 9, 24, and 72 h after the MCAO were 0.58 nM/g (IQR = 0.47–0.70), 0.58 nM/g (IQR = 0.45–0.88), and 0.73 nM/g (IQR = 0.52–0.95), respectively.

In the group of animals treated with glibenclamide, a 64% (1.19 nM/g, IQR = 0.84–1.41) increase in NO levels vs. the control was observed on the third day post-operation (Mann–Whitney test, *p* = 0.0005). Diazoxide administration 1 day before MCAO resulted in a decrease of NO levels at all time points by 21–56%, while statistically significant difference from the control group was observed for samples taken 72 h after MCAO [0.32 nM/g (IQR = 0.25–0.58); Mann–Whitney test, *p* = 0.002].

## Discussion

In preconditioning, there are two phases in the development of the protective effect: the acute phase and the delayed phase, which may differ by the molecular basis of this effect (Nandagopal et al., 2001). Contradictory opinions exist regarding which of the phases plays the major role in the development of cerebral “ischemic tolerance.” Some authors attribute the predominant role to the delayed phase, unlike the heart preconditioning, where defense mechanisms are triggered rather rapidly (Barone et al., 1998). Other researchers observed the emergence of cerebral tolerance to subsequent global ischemia as early as in half an hour after preconditioning (Perez-Pinzon et al., 1997). However, the protective effect of the acute preconditioning phase is believed to be short termed and only capable of delaying cell death (Kirino, 2002).

We found that the density of mitochondrial ${\text{K}}_{\text{ATP}}^{+}$-channel subunit Kir1.1 (KCNJ1 staining) is reduced considerably after ischemic brain preconditioning (Figure 3B). This data explains why there is no reduction in the inner mitochondrial membrane potential in preconditioned cells in ischemia (Kim et al., 2006; Katakam et al., 2007). Another recent study reports that a 2-fold decrease in SUR2 regulatory subunit mRNA expression is observed 48 h after remote ischemic brain post-conditioning (Ezzati et al., 2016). Interestingly, an isoflurane anesthesia was applied for surgical procedures, which itself results in ${\text{K}}_{\text{ATP}}^{+}$-channels opening (Jiang et al., 2007; Swyers et al., 2014) and therefore might be a trigger of the subsequent SUR2 downregulation. Thus, a decrease in mitochondrial ${\text{K}}_{\text{ATP}}^{+}$-channels expression is observed after the mild ischemia/reperfusion or after pharmacological opening of these channels. Can it be the same mechanism that both downregulates mitochondrial ${\text{K}}_{\text{ATP}}^{+}$-channels expression after preconditioning and causes ${\text{K}}_{\text{ATP}}^{+}$-channels dysfunction with cardioprotective deficit in diabetic myocardium (Hassouna et al., 2006)? In opposite to mitochondrial ${\text{K}}_{\text{ATP}}^{+}$-channels, it was recently found that after hypoxic preconditioning in hippocampal neurons there is an increase in activity and expression of the plasmalemmal Kir6.2 subunit (Sun et al., 2015), which prevents membrane excitability and seems to be differentially regulated by the preconditioning impulse. Under severe ischemia myocardial Kir6.2 channels are removed by endocytosis due to phosphorylation by calcium/calmodulin-dependent protein kinase II (CaMKII; Gao et al., 2016). However, the way of how the mitochondrial ${\text{K}}_{\text{ATP}}^{+}$-channel becomes inactive in response to damaging factors remains to be evaluated.

The increase in cytochrome c oxidase density (COX1 staining) was detected in the delayed phase of IP (Figure 3A). Cytochrome c oxidase is known to be a negative regulator of free NO concentration (Torres et al., 2000; Antunes et al., 2007). The neuroprotective effect of the delayed preconditioning phase may persist for days or even weeks (Perez-Pinzon et al., 1997; Nandagopal et al., 2001), and this is consistent with the extraordinary stability of this enzyme (Saikumar and Kurup, 1985). This upregulation is consistent with previously published proteomic data (Cabrera et al., 2012), northern blot and reverse transcription–polymerase chain reaction results (McLeod et al., 2004) that demonstrated increased cytochrome c oxidase and succinate dehydrogenase mRNA expression in the context of delayed ischemic preconditioning. We found no difference in SDHA protein expression between the preconditioned animals and the control group.

Neither the difference in mitochondrial and overall nNOS fluorescence intensity between the groups nor positive staining of iNOS were observed in the preconditioned brain tissue. These results indicate that enzymatic NO synthesis in the delayed IP phase is not regulated at the level of gene expression. In IP models NO synthesis can be suppressed by CaMKII-dependent negative regulatory phosphorylation of nNOS (Wang et al., 2010, 2016). However, ${\text{K}}_{\text{ATP}}^{+}$-channels openers diazoxide and BMS-191095 may initiate NO production by increasing nNOS and eNOS activity due to the activation of positive regulatory phosphorylation through the phosphoinositide-3-kinase/AKT serine/threonine kinase 1 (PI3K-Akt) pathway (Katakam et al., 2013, 2016). Mitochondrial NO-synthase is unlikely to be involved in the preconditioning-induced signaling production of NO as ${\text{K}}_{\text{ATP}}^{+}$-channels opening decreases the mitochondrial capacity for Ca^2+^ ions (Ishida et al., 2004) and it is a Ca^2+^-dependent enzyme (Elfering et al., 2002; Dedkova and Blatter, 2009).

Although an insignificant increase in protein S-nitrosylation (Shen and English, 2005) was noticed at 3 h time point, there was the decrease in S-nitrosoCys fluorescence intensity 24-h post-IP (Figure 3C). This data suggests that the protective effect of the delayed phase of IP do no depend anymore on the protein nitrosylation, which in the acute phase of IP leads to preservation of mitochondrial energetics, reduction of cytosolic Ca^2+^ (Sun et al., 2007) and inhibition of the harmful ROS production (Chouchani et al., 2013; de Lima Portella et al., 2015). As early as 3 h post-IP, we observed a decrease in levels of 3-nitroTyr (Figure 3D)—the marker of NO-dependent oxidative stress (Mohiuddin et al., 2006). The above indirectly points to the activation of the antioxidant systems as a result of tissue conditioning and is confirmed by the studies of neuronal survival, in which the pretreatment with diazoxide prevented cell death via antioxidative pathway activation (Virgili et al., 2013; Shukry et al., 2015). Indeed, diazoxide-induced mitochondrial membrane depolarization (Xi et al., 2005; Vadziuk et al., 2010) can lead to uncoupling of mitochondrial respiration and phosphorylation of adenosine diphosphate molecules (Holmuhamedov et al., 2004) and to the moderate production of ROS (Andrukhiv et al., 2006; Katakam et al., 2016), which may stimulate the antioxidant defense. It is worth to mention that the accumulation of 3-nitroTyr marker was mainly tied to nuclear proteins (Figure 2j). Considering the focus of recent publications toward epigenetic regulation of ischemic tolerance (Aune et al., 2015) and the facts that NO activity is involved in inhibition of histone deacethylases (Illi et al., 2009) and activation of DNA damage repair (Bartz et al., 2015), we hypothesize that delayed neuroprotection caused by IP can be triggered by such ROS as peroxynitrite and nitric oxide (IV), which seems to have important roles in epigenetic regulation of gene expression.

EPR study detected a 2-fold decrease of free NO level (Figure 4) in cerebral cortex post-MCAO, which can be attributed to cerebral hypoperfusion. According to literature reports (Tominaga et al., 1994; Chen et al., 2002), a significant increase in the cerebral EPR signal is observed in MCAO models with spin trap 15 min after vessel occlusion. However, this effect is reversible: NO levels decrease gradually and reach the background values over time (Yuan et al., 2010). Under the IP impulse the production of NO in low-oxygen conditions can be mediated by non-enzymatic nitrite (${\text{NO}}_{2}^{-}$) reduction. ${\text{K}}_{\text{ATP}}^{+}$-channels opening and an increase in K^+^ flow is followed by intense accumulation of osmotically obligate water which results in mitochondrial swelling (Lim et al., 2002). In turn, an increase in matrix volume activates fatty acid β-oxidation (Halestrap, 1987), nicotinamide adenine dinucleotide (NADH) being one of the products of the process (Houten and Wanders, 2010). NADH is an electron donor for the electron transfer network and for molybdenum-containing xanthine oxidoreductase metalloenzyme capable of disoxidating ${\text{NO}}_{2}^{-}$ to NO (Li et al., 2001). Apart from xanthine oxidoreductase, there are several other metalloproteins demonstrating ${\text{NO}}_{2}^{-}$ reductase properties: mitochondrial aldehyde dehydrogenase (Golwala et al., 2009; Perlman et al., 2009), cytochrome c (Basu et al., 2008), neuroglobin (Tiso et al., 2011; Tejero et al., 2015), and cytoglobin (Li et al., 2012). It is possible that their combined effect leads to the appearance of signaling NO production.

We demonstrated that PhP with ${\text{K}}_{\text{ATP}}^{+}$-channels opener diazoxide causes a 2-fold reduction of free NO concentration 72 h post-MCAO. This relationship can be mediated by the enhancement of cytochrome c oxidase function after the preconditioning. Although it is reported that ischemia-associated signaling production of NO (Tominaga et al., 1994; Chen et al., 2002) results in inhibition of oxygen consumption by cytochrome c oxidase (Brunori et al., 2004; Palacios-Callender et al., 2007; Sarti et al., 2012), there are two possible ways of NO interaction. NO binds to metal ions in the active center of cytochrome c oxidase to produce either nitrosyl- or nitrite derivatives (Gibson and Greenwood, 1963; Brudvig et al., 1980). These reactions dominate over each other depending on the oxygen concentration and the electron flow in the respiratory chain (Sarti et al., 2000). In the first period after the preconditioning impulse a proportion of cytochrome c oxidase subunits is likely be found in a reduced state. Association with a reduced form of the enzyme under ischemia conditions is rapid and followed by a longterm inhibition of cell respiration until the dissociation of NO, while its interaction with an oxidized form of the enzyme initiates 1,000 times slower, has 10–20 times shorter duration and results in a production of ${\text{NO}}_{2}^{-}$ (Giuffrè et al., 2000). In the delayed preconditioning phase, against the background of the increase in cytochrome c oxidase density, most of the enzyme molecules will be oxidized which creates favorable conditions for the second scenario of NO oxidation to ${\text{NO}}_{2}^{-}$, therefore reducing NO bioavailability and diminishing its damaging effect.

There was a sharp increase in cerebral tissue NO on the third day after stroke in the group of animals treated with glibenclamide immediately prior to MCAO (Figure 4). We associate the obtained result with the activation of iNOS, the level of which increases in the tissues in response to the inflammation. According to literature, the expression of microglial cell membrane ${\text{K}}_{\text{ATP}}^{+}$-channels is increased in response to pro-inflammatory signals, and exposure to glibenclamide enhances the extent of microglial cell activation considerably (Ortega et al., 2012) which results in increased cytokine, NO, and ROS production (Wang et al., 2004).

In conclusion, the opening of ${\text{K}}_{\text{ATP}}^{+}$-channels, which initiates a change in the mitochondrial matrix ionic composition and triggers the protective mechanisms, plays a key role in the development of preconditioning phenomenon. Mild ischemia/reperfusion leads to the reduction in density of mitochondrial ${\text{K}}_{\text{ATP}}^{+}$-channels that prevents a drop in the inner mitochondrial membrane potential in post-ischemic preconditioning period. The amount of protein nitrosylation and nitration is also lower in preconditioned tissue. We observed the decrease in NO cerebral content in the MCAO model after PhP with ${\text{K}}_{\text{ATP}}^{+}$-channels opener diazoxide and attribute it to the enhanced ability of the overexpressed cytochrome c oxidase to consume free NO, thereby preventing the reduction of mitochondrial sensitivity to oxygen in the period of severe ischemia. In addition to their involvement in the preconditioning process, plasmalemmal ${\text{K}}_{\text{ATP}}^{+}$-channels appear to play a special role in the regulation of the intensity of NO production and microglial inflammation in ischemic cerebral tissue.

Further research is required to study the feedback mechanisms of the changes in expression of key mitochondrial energy metabolism molecules in response to signaling production of ROS and NO—the unconditional mediators of preconditioning phenomenon.

## Author contributions

OD, SG, and AG performed the experimental manipulations with the laboratory animals. OD and SB contributed to the acquisition and analysis of IHC data. KG, VA, and GY contributed to the acquisition and analysis of EPR data. SG and OD designed the experiments, interpreted the data and wrote the manuscript. VK, SB, and KG provided supervision, critical revision of the article, and final approval.

### Conflict of interest statement

The authors declare that the research was conducted in the absence of any commercial or financial relationships that could be construed as a potential conflict of interest. The reviewer LG and handling Editor declared their shared affiliation, and the handling Editor states that the process nevertheless met the standards of a fair and objective review

## References

[B1] Andrukhiv A. Costa A. D. West I. C. Garlid K. D. (2006). Opening mitoKATP increases superoxide generation from complex I of the electron transport chain. Am. J. Physiol. Heart Circ. Physiol. 291, 2067–2074. 10.1152/ajpheart.00272.2006 16798828

[B2] Antunes F. Boveris A. Cadenas E. (2007). On the biologic role of the reaction of NO with oxidized cytochrome coxidase. Antioxid. Redox Signal. 9, 1569–1579. 10.1089/ars.2007.1677 17665969

[B3] Aune S. E. Herr D. J. Kutz C. J. Menick D. R. (2015). Histone deacetylases exert class-specific roles in conditioning the brain and heart against acute ischemic injury. Front. Neurol. 6:e145. 10.3389/fneur.2015.00145 26175715 PMC4485035

[B4] Bajgar R. Seetharaman S. Kowaltowski A. J. Garlid K. D. Paucek P. (2001). Identification and properties of a novel intracellular (mitochondrial) ATP-sensitive potassium channel in brain. J. Biol. Chem. 276, 33369–33374. 10.1074/jbc.M103320200 11441006

[B5] Barone F. C. White R. F. Spera P. A. Ellison J. Currie R. W. Wang X.. (1998). Ischemic preconditioning and brain tolerance: temporal histological and functional outcomes, protein synthesis requirement, and interleukin-1 receptor antagonist and early gene expression. Stroke 29, 1937–1951. 10.1161/01.STR.29.9.1937 9731622

[B6] Barr D. Jiang J. Weber R. T. (2000). How to Quantitate Nitroxide Spin Adducts Using TEMPOL. EPR Division, Bruker, SpinReport, 3–6.

[B7] Bartz R. R. Suliman H. B. Piantadosi C. A. (2015). Redox mechanisms of cardiomyocyte mitochondrial protection. Front. Physiol. 6:e291. 10.3389/fphys.2015.00291 26578967 PMC4620408

[B8] Basu S. Azarova N. A. Font M. D. King S. B. Hogg N. Gladwin M. T.. (2008). Nitrite reductase activity of cytochrome C. J. Biol. Chem. 283, 32590–32597. 10.1074/jbc.M806934200 18820338 PMC2583304

[B9] Bolanos J. P. Almeida A. (1999). Roles of nitric oxide in brain hypoxia-ischemia. Biochim. Biophys. Acta 1411, 415–436. 10.1016/S0005-2728(99)00030-4 10320673

[B10] Brown G. C. Cooper C. E. (1994). Nanomolar concentrations of nitric oxide reversibly inhibit synaptosomal respiration by competing with oxygen at cytochrome oxidase. FEBS Lett. 356, 295–298. 10.1016/0014-5793(94)01290-3 7805858

[B11] Brudvig G. W. Stevens T. H. Chan S. I. (1980). Reactions of nitric oxide with cytochrome c oxidase. Biochemistry 19, 5275–5285. 10.1021/bi00564a020 6255988

[B12] Brunori M. Giuffrè A. Forte E. Mastronicola D. Barone M. C. Sarti P. (2004). Control of cytochrome c oxidase activity by nitric oxide. Biochim. Biophys. Acta 1655, 365–371. 10.1016/j.bbabio.2003.06.008 15100052

[B13] Brustovetsky T. Shalbuyeva N. Brustovetsky N. (2005). Lack of manifestations of diazoxide/5-hydroxydecanoate-sensitive KATP channel in rat brain nonsynaptosomal mitochondria. J. Physiol. 568, 47–59. 10.1113/jphysiol.2005.091199 16051627 PMC1474777

[B14] Cabrera J. A. Ziemba E. A. Colbert R. Anderson L. B. Sluiter W. Duncker D. J.. (2012). Altered expression of mitochondrial electron transport chain proteins and improved myocardial energetic state during late ischemic preconditioning. Am. J. Physiol. Heart Circ. Physiol. 302, H1974–H1982. 10.1152/ajpheart.00372.2011 22389388 PMC3362109

[B15] Calabrese E. J. (2016). Pre- and post-conditioning hormesis in elderly mice, rats, and humans: its loss and restoration. Biogerontology 17, 681–702. 10.1007/s10522-016-9646-8 27075594

[B16] Chen S. H. Fung P. C. Cheung R. T. (2002). Neuropeptide Y-Y1 receptor modulates nitric oxide level during stroke in the rat. Free Radic. Biol. Med. 32, 776–784. 10.1016/S0891-5849(02)00774-8 11937303

[B17] Chouchani E. T. Methner C. Nadtochiy S. M. Logan A. Pell V. R. Ding S.. (2013). Cardioprotection by S-nitrosation of a cysteine switch on mitochondrial complex I. Nat. Med. 19, 753–759. 10.1038/nm.3212 23708290 PMC4019998

[B18] Correia S. C. Carvalho C. Cardoso S. Santos R. X. Santos M. S. Oliveira C. R.. (2010). Mitochondrial preconditioning: a potential neuroprotective strategy. Front. Aging Neurosci. 2:e138. 10.3389/fnagi.2010.00138 20838473 PMC2936931

[B19] Cuomo O. Vinciguerra A. Cerullo P. Anzilotti S. Brancaccio P. Bilo L.. (2015). Ionic homeostasis in brain conditioning. Front. Neurosci. 9:e277. 10.3389/fnins.2015.00277 26321902 PMC4530315

[B20] Dedkova E. N. Blatter L. A. (2009). Characteristics and function of cardiac mitochondrial nitric oxide synthase. J. Physiol. 587, 851–872. 10.1113/jphysiol.2008.165423 19103678 PMC2669975

[B21] de Lima Portella R. Lynn Bickta J. Shiva S. (2015). Nitrite confers preconditioning and cytoprotection after ischemia/reperfusion injury through the modulation of mitochondrial function. Antioxid. Redox Signal. 23, 307–327. 10.1089/ars.2015.6260 26094636

[B22] Deryagin O. G. Gavrilova S. A. Buravkov S. V. Andrianov V. V. Yafarova G. G. Gainutdinov Kh. L. (2016). The role of ATP-dependent potassium channels and nitric oxide system in the neuroprotective effect of preconditioning. Zh. Nevrol. Psikhiatr. Im. S. S. Korsakova 8, 16–22. 10.17116/jnevro20161168217-23 27905383

[B23] Ding Z. M. Wu B. Zhang W. Q. Lu X. J. Lin Y. C. Geng Y. J.. (2012). Neuroprotective effects of ischemic preconditioning and postconditioning on global brain ischemia in rats through the same effect on inhibition of apoptosis. Int. J. Mol. Sci. 13, 6089–6101. 10.3390/ijms13056089 22754351 PMC3382765

[B24] Dröse S. (2013). Differential effects of complex II on mitochondrial ROS production and their relation to cardioprotective pre- and postconditioning. Biochim. Biophys. Acta 1827, 578–587. 10.1016/j.bbabio.2013.01.004 23333272

[B25] Elfering S. L. Sarkela T. M. Giulivi C. (2002). Biochemistry of mitochondrial nitric-oxide synthase. J. Biol. Chem. 277, 38079–38086. 10.1074/jbc.M205256200 12154090

[B26] Ezzati M. Bainbridge A. Broad K. D. Kawano G. Oliver-Taylor A. Rocha-Ferreira E. (2016). Immediate remote ischemic postconditioning after hypoxia ischemia in piglets protects cerebral white matter but not grey matter. J. Cereb. Blood Flow Metab. 36, 1396–1411. 10.1177/0271678X15608862 26661194 PMC4976661

[B27] Foster D. B. Ho A. S. Rucker J. Garlid A. O. Chen L. Sidor A.. (2012). Mitochondrial ROMK channel is a molecular component of mitoK(ATP). Circ. Res. 111, 446–454. 10.1161/CIRCRESAHA.112.266445 22811560 PMC3560389

[B28] Foster D. B. Rucker J. J. Marbán E. (2008). Is Kir6.1 a subunit of mitoK(ATP)? Biochem. Biophys. Res. Commun. 366, 649–656. 10.1016/j.bbrc.2007.11.154 18068667 PMC2276631

[B29] Foster M. N. Coetzee W. A. (2016). KATP channels in the cardiovascular system. Physiol. Rev. 96, 177–252. 10.1152/physrev.00003.2015 26660852 PMC4698399

[B30] Gainutdinov Kh. L. Andrianov V. V. Iyudin V. S. Yurtaeva S. V. Jafarova G. G. Faisullina R. I. (2013). EPR study of nitric oxide production in rat tissues under hypokinesia. Biophysics 58, 203–205. 10.1134/S0006350913020073

[B31] Gainutdinov Kh. L. Gavrilova S. A. Iyudin V. S. Golubeva A. V. Davydova M. P. Jafarova G. G. (2011). EPR study of the intensity of the nitric oxide production in rat brain after ischemic stroke. Appl. Magn. Reson. 40, 267–278. 10.1007/s00723-011-0207-7

[B32] Gao Z. Sierra A. Zhu Z. Koganti S. R. Subbotina E. Maheshwari A. (2016). Loss of ATP-sensitive potassium channel surface expression in heart failure underlies dysregulation of action potential duration and myocardial vulnerability to injury. PLoS ONE 11:e0151337. 10.1371/journal.pone.0151337 26964104 PMC4786327

[B33] Garlid A. O. Jabůrek M. Jacobs J. P. Garlid K. D. (2013). Mitochondrial reactive oxygen species: which ROS signals cardioprotection? Am. J. Physiol. Heart Circ. Physiol. 305, 960–968. 10.1152/ajpheart.00858.2012 23913710 PMC3798754

[B34] Gibson Q. H. Greenwood C. (1963). Reactions of cytochrome oxidase with oxygen and carbon monoxide. Biochem. J. 86, 541–554. 10.1042/bj0860541 13947736 PMC1201788

[B35] Giuffrè A. Barone M. C. Mastronicola D. D'Itri E. Sarti P. Brunori M. (2000). Reaction of nitric oxide with the turnover intermediates of cytochrome c oxidase: reaction pathway and functional effects. Biochemistry 39, 15446–15453. 10.1021/bi000447k 11112530

[B36] Golwala N. H. Hodenette C. Murthy S. N. Nossaman B. D. Kadowitz P. J. (2009). Vascular responses to nitrite are mediated by xanthine oxidoreductase and mitochondrial aldehyde dehydrogenase in the rat. Can. J. Physiol. Pharmacol. 87, 1095–1101. 10.1139/Y09-101 20029546

[B37] Guo D. Nguyen T. Ogbi M. Tawfik H. Ma G. Yu Q.. (2007). Protein kinase C-epsilon coimmunoprecipitates with cytochrome oxidase subunit IV and is associated with improved cytochrome-c oxidase activity and cardioprotection. Am. J. Physiol. Heart Circ. Physiol. 293, H2219–H2230. 10.1152/ajpheart.01306.2006 17660387

[B38] Halestrap A. P. (1987). The regulation of oxidation of fatty acids and other substrates in rat heart mitochondria by changes in the matrix volume induced by osmotic strength, valinomycin and Ca^2+^. Biochem. J. 244, 159–164. 10.1042/bj2440159 3663110 PMC1147967

[B39] Hassouna A. Loubani M. Matata B. M. Fowler A. Standen N. B. Galiñanes M. (2006). Mitochondrial dysfunction as the cause of the failure to precondition the diabetic human myocardium. Cardiovasc. Res. 69, 450–458. 10.1016/j.cardiores.2005.11.004 16330008

[B40] Holmuhamedov E. L. Jahangir A. Oberlin A. Komarov A. Colombini M. Terzic A. (2004). Potassium channel openers are uncoupling protonophores: implication in cardioprotection. FEBS Lett. 568, 167–170. 10.1016/j.febslet.2004.05.031 15196941

[B41] Houten S. M. Wanders R. J. (2010). A general introduction to the biochemistry of mitochondrial fatty acid β-oxidation. J. Inherit. Metab. Dis. 33, 469–477. 10.1007/s10545-010-9061-2 20195903 PMC2950079

[B42] Illi B. Colussi C. Grasselli A. Farsetti A. Capogrossi M. C. Gaetano C. (2009). NO sparks off chromatin: tales of a multifaceted epigenetic regulator. Pharmacol. Ther. 123, 344–352. 10.1016/j.pharmthera.2009.05.003 19464317

[B43] Ishida H. Higashijima N. Hirota Y. Genka C. Nakazawa H. Nakaya H.. (2004). Nicorandil attenuates the mitochondrial Ca^2+^ overload with accompanying depolarization of the mitochondrial membrane in the heart. Naunyn Schmiedebergs. Arch. Pharmacol. 369, 192–197. 10.1007/s00210-003-0851-z 14685646

[B44] Jabůrek M. Costa A. D. Burton J. R. Costa C. L. Garlid K. D. (2006). Mitochondrial PKC epsilon and mitochondrial ATP-sensitive K^+^ channel copurify and coreconstitute to form a functioning signaling module in proteoliposomes. Circ. Res. 99, 878–883. 10.1161/01.RES.0000245106.80628.d3 16960097

[B45] Jiang M. T. Nakae Y. Ljubkovic M. Kwok W. M. Stowe D. F. Bosnjak Z. J. (2007). Isoflurane activates human cardiac mitochondrial adenosine triphosphate-sensitive K+ channels reconstituted in lipid bilayers. Anesth. Analg. 105, 926–932. 10.1213/01.ane.0000278640.81206.92 17898367

[B46] Jung K. H. Chu K. Ko S. Y. Lee S. T. Sinn D. I. Park D. K.. (2006). Early intravenous infusion of sodium nitrite protects brain against *in vivo* ischemia-reperfusion injury. Stroke 37, 2744–2750. 10.1161/01.STR.0000245116.40163.1c 17008610

[B47] Kalogeris T. Bao Y. Korthuis R. J. (2014). Mitochondrial reactive oxygen species: a double edged sword in ischemia/reperfusion vs preconditioning. Redox Biol. 2, 702–714. 10.1016/j.redox.2014.05.006 24944913 PMC4060303

[B48] Katakam P. V. Dutta S. Sure V. N. Grovenburg S. M. Gordon A. O. Peterson N. R.. (2016). Depolarization of mitochondria in neurons promotes activation of nitric oxide synthase and generation of nitric oxide. Am. J. Physiol. Heart Circ. Physiol. 310, H1097–H1106. 10.1152/ajpheart.00759.2015 26945078 PMC4867394

[B49] Katakam P. V. Jordan J. E. Snipes J. A. Tulbert C. D. Miller A. W. Busija D. W. (2007). Myocardial preconditioning against ischemia-reperfusion injury is abolished in Zucker obese rats with insulin resistance. Am. J. Physiol. Regul. Integr. Comp. Physiol. 292, R920–R926. 10.1152/ajpregu.00520.2006 17008456

[B50] Katakam P. V. Wappler E. A. Katz P. S. Rutkai I. Institoris A. Domoki F.. (2013). Depolarization of mitochondria in endothelial cells promotes cerebral artery vasodilation by activation of nitric oxide synthase. Arterioscler. Thromb. Vasc. Biol. 33, 752–759. 10.1161/ATVBAHA.112.300560 23329133 PMC3628534

[B51] Kim M. Y. Kim M. J. Yoon I. S. Ahn J. H. Lee S. H. Baik E. J.. (2006). Diazoxide acts more as a PKC-epsilon activator, and indirectly activates the mitochondrial K(ATP) channel conferring cardioprotection against hypoxic injury. Br. J. Pharmacol. 149, 1059–1070. 10.1038/sj.bjp.0706922 17043673 PMC2014640

[B52] Kirino T. (2002). Ischemic tolerance. J. Cereb. Blood Flow Metab. 22, 1283–1296. 10.1097/01.WCB.0000040942.89393.88 12439285

[B53] Kitamura Y. Matsuoka Y. Nomura Y. Taniguchi T. (1998). Induction of inducible nitric oxide synthase and heme oxygenase-1 in rat glial cells. Life Sci. 62, 1717–1721. 10.1016/S0024-3205(98)00134-9 9585163

[B54] Konstas A. A. Dabrowski M. Korbmacher C. Tucker S. J. (2002). Intrinsic sensitivity of Kir1.1 (ROMK) to glibenclamide in the absence of SUR2B. implications for the identity of the renal ATP-regulated secretory *K*+ channel. J. Biol. Chem. 277, 21346–21351. 10.1074/jbc.M202005200 11927600

[B55] Korichneva I. Hoyos B. Chua R. Levi E. Hammerling U. (2002). Zinc release from protein kinase C as the common event during activation by lipid second messenger or reactive oxygen. J. Biol. Chem. 277, 44327–44331. 10.1074/jbc.M205634200 12213816

[B56] Lacza Z. Snipes J. A. Kis B. Szabó C. Grover G. Busija D. W. (2003a). Investigation of the subunit composition and the pharmacology of the mitochondrial ATP-dependent K+ channel in the brain. Brain Res. 994, 27–36. 10.1016/j.brainres.2003.09.046 14642445

[B57] Lacza Z. Snipes J. A. Miller A. W. Szabó C. Grover G. Busija D. W. (2003b). Heart mitochondria contain functional ATP-dependent K^+^ channels. J. Mol. Cell. Cardiol. 35, 1339–1347. 10.1016/S0022-2828(03)00249-9 14596790

[B58] Li H. Hemann C. Abdelghany T. M. El-Mahdy M. A. Zweier J. L. (2012). Characterization of the mechanism and magnitude of cytoglobin-mediated nitrite reduction and nitric oxide generation under anaerobic conditions. J. Biol. Chem. 287, 36623–36633. 10.1074/jbc.M112.342378 22896706 PMC3476328

[B59] Li H. Samouilov A. Liu X. Zweier J. L. (2001). Characterization of the magnitude and kinetics of xanthine oxidase-catalyzed nitrite reduction. Evaluation of its role in nitric oxide generation in anoxic tissues. J. Biol. Chem. 276, 24482–24489. 10.1074/jbc.M011648200 11312267

[B60] Lim K. H. Javadov S. A. Das M. Clarke S. J. Suleiman M. S. Halestrap A. P. (2002). The effects of ischaemic preconditioning, diazoxide and 5-hydroxydecanoate on rat heart mitochondrial volume and respiration. J. Physiol. 545, 961–974. 10.1113/jphysiol.2002.031484 12482899 PMC2290722

[B61] Lim S. Y. Hausenloy D. J. (2012). Remote ischemic conditioning: from bench to bedside. Front. Physiol. 3:e27. 10.3389/fphys.2012.00027 22363297 PMC3282534

[B62] Liu D. Lu C. Wan R. Auyeung W. W. Mattson M. P. (2002). Activation of mitochondrial ATP-dependent potassium channels protects neurons against ischemia-induced death by a mechanism involving suppression of Bax translocation and cytochrome c release. J. Cereb. Blood Flow Metab. 22, 431–443. 10.1097/00004647-200204000-00007 11919514

[B63] Madungwe N. B. Zilberstein N. F. Feng Y. Bopassa J. C. (2016). Critical role of mitochondrial ROS is dependent on their site of production on the electron transport chain in ischemic heart. Am. J. Cardiovasc. Dis. 6, 93–108. 27679744 PMC5030389

[B64] Manukhina E. B. Malyshev I. Y. Smirin B. V. Mashina S. Y. Saltykova V. A. Vanin A. F. (1999). Production and storage of nitric oxide in adaptation to hypoxia. Nitric Oxide 3, 393–401. 10.1006/niox.1999.0244 10534443

[B65] Marshall J. M. Thomas T. Turner L. (1993). A link between adenosine, ATP-sensitive K^+^ channels, potassium and muscle vasodilatation in the rat in systemic hypoxia. J. Physiol. 472, 1–9. 10.1113/jphysiol.1993.sp019931 8145135 PMC1160471

[B66] Maslov L. N. Khaliulin I. G. Podoksenov Yu. K. (2012). Neuroprotective and cardioprotective effects of hypothermic preconditioning. Patol. Fiziol. Eksp. Ter. 1, 67–72 22629865

[B67] McLeod C. J. Jeyabalan A. P. Minners J. O. Clevenger R. Hoyt R. F. Jr. Sack M. N. (2004). Delayed ischemic preconditioning activates nuclear-encoded electron-transfer-chain gene expression in parallel with enhanced postanoxic mitochondrial respiratory recovery. Circulation 110, 534–539. 10.1161/01.CIR.0000136997.53612.6C 15277332

[B68] Mikoyan V. D. Kubrina L. N. Serezhenkov V. A. Stukan R. A. Vanin A. F. (1997). Complexes of Fe^2+^ with diethyldithiocarbamate or N-methyl-D-glucamine dithiocarbamate as traps of nitric oxide in animal tissues. Biochim. Biophys. Acta 1336, 225–2340. 10.1016/S0304-4165(97)00032-9 9305794

[B69] Mironova G. D. Kachaeeva E. V. Krylova I. B. Rodionova O. M. Balina M. I. Evdokimova N. R. (2007). Mitochondrial ATP-dependent potassium channel. 2. The role of the channel in protection of the heart against ischemia. Vestn. Ross. Akad. Med. Nauk. 2, 44–49. 17396562

[B70] Mironova G. D. Negoda A. E. Marinov B. S. Paucek P. Costa A. D. Grigoriev S. M.. (2004). Functional distinctions between the mitochondrial ATP-dependent K+ channel (mitoKATP) and its inward rectifier subunit (mitoKIR). J. Biol. Chem. 279, 32562–32568. 10.1074/jbc.M401115200 15138282

[B71] Mohiuddin I. Chai H. Lin P. H. Lumsden A. B. Yao Q. Chen C. (2006). Nitrotyrosine and chlorotyrosine: clinical significance and biological functions in the vascular system. J. Surg. Res. 133, 143–149. 10.1016/j.jss.2005.10.008 16360172

[B72] Murata M. Akao M. O'Rourke B. Marbán E. (2001). Mitochondrial ATP-sensitive potassium channels attenuate matrix Ca^2+^ overload during simulated ischemia and reperfusion: possible mechanism of cardioprotection. Circ. Res. 89, 891–898. 10.1161/hh2201.100205 11701616

[B73] Nandagopal K. Dawson T. M. Dawson V. L. (2001). Critical role for nitric oxide signaling in cardiac and neuronal ischemic preconditioning and tolerance. J. Pharmacol. Exp. Ther. 297, 474–478. 11303032

[B74] Ortega F. J. Gimeno-Bayon J. Espinosa-Parrilla J. F. Carrasco J. L. Batlle M. Pugliese M.. (2012). ATP-dependent potassium channel blockade strengthens microglial neuroprotection after hypoxia-ischemia in rats. Exp. Neurol. 235, 282–296. 10.1016/j.expneurol.2012.02.010 22387180

[B75] Palacios-Callender M. Hollis V. Mitchison M. Frakich N. Unitt D. Moncada S. (2007). Cytochrome c oxidase regulates endogenous nitric oxide availability in respiring cells: a possible explanation for hypoxic vasodilation. Proc. Natl. Acad. Sci. U.S.A. 104, 18508–18513. 10.1073/pnas.0709440104 18003892 PMC2141807

[B76] Perez-Pinzon M. A. Xu G. P. Dietrich W. D. Rosenthal M. Sick T. J. (1997). Rapid preconditioning protects rats against ischemic neuronal damage after 3 but not 7 days of reperfusion following global cerebral ischemia. J. Cereb. Blood Flow Metab. 17, 175–182. 10.1097/00004647-199702000-00007 9040497

[B77] Perlman D. H. Bauer S. M. Ashrafian H. Bryan N. S. Garcia-Saura M. F. Lim C. C.. (2009). Mechanistic insights into nitrite-induced cardioprotection using an integrated metabolomic/proteomic approach. Circ. Res. 104, 796–804. 10.1161/CIRCRESAHA.108.187005 19229060 PMC6731772

[B78] Rana A. Goyal N. Ahlawat A. Jamwal S. Reddy B. V. Sharma S. (2015). Mechanisms involved in attenuated cardio-protective role of ischemic preconditioning in metabolic disorders. Perfusion 30, 94–105. 10.1177/0267659114536760 24947460

[B79] Rybnikova E. Samoilov M. (2015). Current insights into the molecular mechanisms of hypoxic pre- and postconditioning using hypobaric hypoxia. Front. Neurosci. 9:e388. 10.3389/fnins.2015.00388 26557049 PMC4615940

[B80] Saikumar P. Kurup C. K. (1985). Effect of administration of 2-methyl-4-dimethylaminoazobenzene on the half-lives of rat liver mitochondria and cytochrome oxidase. Biochim. Biophys. Acta 840, 127–133. 10.1016/0304-4165(85)90169-2 2986708

[B81] Samoilenkova N. S. Gavrilova S. A. Dubina A. I. Khudoerkov R. M. Pirogov Ju. A. (2007). Role of ATP-sensitive potassium channel in hypoxic and ischemic types of preconditioning in rat brain with focal ischemia. Regionarnoe Krovoobrashchenie i Mikrotsirkulyatsiya 6, 68–77.

[B82] Samoilenkova N. S. Gavrilova S. A. Koshelev V. B. (2008). Neuroprotective and angioprotective effect of ischemic/hypoxic preconditioning of the brain. Regionarnoe Krovoobrashchenie i Mikrotsirkulyatsiya 1, 82–92.

[B83] Sarti P. Forte E. Mastronicola D. Giuffrè A. Arese M. (2012). Cytochrome coxidase and nitric oxide in action: molecular mechanisms and pathophysiological implications. Biochim. Biophys. Acta 1817, 610–619. 10.1016/j.bbabio.2011.09.002 21939634

[B84] Sarti P. Giuffré A. Forte E. Mastronicola D. Barone M. C. Brunori M. (2000). Nitric oxide and cytochrome c oxidase: mechanisms of inhibition and NO degradation. Biochem. Biophys. Res. Commun. 274, 183–187. 10.1006/bbrc.2000.3117 10903916

[B85] Sasaki N. Sato T. Ohler A. O'Rourke B. Marbán E. (2000). Activation of mitochondrial ATP-dependent potassium channels by nitric oxide. Circulation 101, 439–445. 10.1161/01.CIR.101.4.439 10653837

[B86] Schulz R. Ferdinandy P. (2013). Does nitric oxide signaling differ in pre- and post-conditioning? Importance of S-nitrosylation vs. protein kinase G activation. Free Radic. Biol. Med. 54, 113–115. 10.1016/j.freeradbiomed.2012.10.547 23089225

[B87] Shen B. English A. M. (2005). Mass spectrometric analysis of nitroxyl-mediated protein modification: comparison of products formed with free and protein-based cysteines. Biochemistry 44, 14030–14044. 10.1021/bi0507478 16229492

[B88] Shimizu K. Lacza Z. Rajapakse N. Horiguchi T. Snipes J. Busija D. W. (2002). MitoK(ATP) opener, diazoxide, reduces neuronal damage after middle cerebral artery occlusion in the rat. Am. J. Physiol. Heart Circ. Physiol. 283, H1005–H1011. 10.1152/ajpheart.00054.2002 12181130

[B89] Shmonin A. A. Baisa A. E. Melnikova E. V. Vavilov V. N. Vlasov T. D. (2012). Protective effects of early ischemic preconditioning in focal cerebral ischemia in rats: the role of collateral blood circulation. Neurosci. Behav. Physiol. 42, 643–650. 10.1007/s11055-012-9615-x

[B90] Shukry M. Kamal T. Ali R. Farrag F. Almadaly E. Saleh A. A.. (2015). Pinacidil and levamisole prevent glutamate-induced death of hippocampal neuronal cells through reducing ROS production. Neurol. Res. 37, 916–923. 10.1179/1743132815Y.0000000077 26183935

[B91] Singh H. Hudman D. Lawrence C. L. Rainbow R. D. Lodwick D. Norman R. I. (2003). Distribution of Kir6.0 and SUR2 ATP-sensitive potassium channel subunits in isolated ventricular myocytes. J. Mol. Cell. Cardiol. 35, 445–459. 10.1016/S0022-2828(03)00041-5 12738227

[B92] Sun H. S. Xu B. Chen W. Xiao A. Turlova E. Alibraham A. (2015). Neuronal K(ATP) channels mediate hypoxic preconditioning and reduce subsequent neonatal hypoxic-ischemic brain injury. Exp. Neurol. 263, 161–171. 10.1016/j.expneurol.2014.10.003 25448006

[B93] Sun J. Morgan M. Shen R. F. Steenbergen C. Murphy E. (2007). Preconditioning results in S-nitrosylation of proteins involved in regulation of mitochondrial energetics and calcium transport. Circ. Res. 101, 1155–1163. 10.1161/CIRCRESAHA.107.155879 17916778

[B94] Swyers T. Redford D. Larson D. F. (2014). Volatile anesthetic-induced preconditioning. Perfusion 29, 10–15. 10.1177/0267659113503975 24002781

[B95] Talanov E. Y. Pavlik L. L. Mikheeva I. B. Murzaeva S. V. Ivanov A. N. Mironova G. D. (2016). Ultrastructural localization of the ROMK potassium channel in rat liver and heart. Biochem. Moscow Suppl. Ser. A 10, 195–198. 10.1134/S1990747816020100

[B96] Tejero J. Sparacin.o-Watkins C. E. Ragireddy V. Frizzell S. Gladwin M. T. (2015). Exploring the mechanisms of the reductase activity of neuroglobin by site-directed mutagenesis of the heme distal pocket. Biochemistry 54, 722–733. 10.1021/bi501196k 25554946 PMC4410703

[B97] Terpolilli N. A. Moskowitz M. A. Plesnila N. (2012). Nitric oxide: considerations for the treatment of ischemic stroke. J. Cereb. Blood Flow Metab. 32, 1332–1346. 10.1038/jcbfm.2012.12 22333622 PMC3390820

[B98] Thuret R. Saint Yves T. Tillou X. Chatauret N. Thuillier R. Barrou B. (2014). Ischemic pre- and post-conditioning: current clinical applications. Prog. Urol. 24, S56–S61. 10.1016/s1166-7087(14)70065-x 24950935

[B99] Tiso M. Tejero J. Basu S. Azarov I. Wang X. Simplaceanu V.. (2011). Human neuroglobin functions as a redox-regulated nitrite reductase. J. Biol. Chem. 286, 18277–18289. 10.1074/jbc.M110.159541 21296891 PMC3093900

[B100] Tominaga T. Sato S. Ohnishi T. Ohnishi S. T. (1994). Electron paramagnetic resonance (EPR) detection of nitric oxide produced during forebrain ischemia of the rat. J. Cereb. Blood Flow Metab. 14, 715–722. 10.1038/jcbfm.1994.92 8063867

[B101] Torres J. Sharpe M. A. Rosquist A. Cooper C. E. Wilson M. T. (2000). Cytochrome c oxidase rapidly metabolises nitric oxide to nitrite. FEBS Lett. 475, 263–266. 10.1016/S0014-5793(00)01682-3 10869568

[B102] Vadziuk O. B. Chunikhin O. Kosterin S. O. (2010). Effect of mitochondrial ATP-dependent potassium channel effectors diazoxide and glybenclamide on hydrodynamic diameter and membrane potential of the myometrial mitochondria. Ukr. Biokhim. Zh. 82, 40–47. 21516715

[B103] Vanin A. F. Huisman A. Van Faassen E. E. (2003). Iron dithiocarbamate as spin trap for nitric oxide detection: pitfalls and successes. Methods Enzymol. 359, 27–42. 10.1016/S0076-6879(02)59169-2 12481557

[B104] Virgili N. Mancera P. Wappenhans B. Sorrosal G. Biber K. Pugliese M.. (2013). K(ATP) channel opener diazoxide prevents neurodegeneration: a new mechanism of action via antioxidative pathway activation. PLoS ONE 8:e75189. 10.1371/annotation/0e045706-ea24-41db-be90-27d1cbcd35b1 24040400 PMC3770693

[B105] Wang M. Qi D. S. Zhou C. Han D. Li P. P. Zhang F.. (2016). Ischemic preconditioning protects the brain against injury via inhibiting CaMKII-nNOS signaling pathway. Brain Res. 1634, 140–149. 10.1016/j.brainres.2016.01.008 26794251

[B106] Wang T. Qin L. Liu B. Liu Y. Wilson B. Eling T. E.. (2004). Role of reactive oxygen species in LPS-induced production of prostaglandin E2 in microglia. J. Neurochem. 88, 939–947. 10.1046/j.1471-4159.2003.02242.x 14756815

[B107] Wang W. W. Hu S. Q. Li C. Zhou C. Qi S. H. Zhang G. Y. (2010). Transduced PDZ1 domain of PSD-95 decreases Src phosphorylation and increases nNOS (Ser847) phosphorylation contributing to neuroprotection after cerebral ischemia. Brain Res. 1328, 162–170. 10.1016/j.brainres.2010.02.055 20197063

[B108] Wang Y. Reis C. Applegate R. II. Stier G. Martin R. Zhang J. H. (2015). Ischemic conditioning-induced endogenous brain protection: applications pre-, per- or post-stroke. Exp. Neurol. 272, 26–40. 10.1016/j.expneurol.2015.04.009 25900056 PMC4609591

[B109] Wojtovich A. P. Smith C. O. Haynes C. M. Nehrke K. W. Brookes P. S. (2013). Physiological consequences of complex II inhibition for aging, disease, and the mKATP channel. Biochim. Biophys. Acta 1827, 598–611. 10.1016/j.bbabio.2012.12.007 23291191 PMC3880126

[B110] Xi Q. Cheranov S. Y. Jaggar J. H. (2005). Mitochondria-derived reactive oxygen species dilate cerebral arteries by activating Ca^2+^ sparks. Circ. Res. 97, 354–362. 10.1161/01.RES.0000177669.29525.78 16020754 PMC1352308

[B111] Yamada K. Ji J. J. Yuan H. Miki T. Sato S. Horimoto N.. (2001). Protective role of ATP-sensitive potassium channels in hypoxia-induced generalized seizure. Science 292, 1543–1546. 10.1126/science.1059829 11375491

[B112] Yuan Z. Liu W. Liu B. Schnell A. Liu K. J. (2010). Normobaric hyperoxia delays and attenuates early nitric oxide production in focal cerebral ischemic rats. Brain Res. 1352, 248–254. 10.1016/j.brainres.2010.07.010 20633543 PMC2930605

[B113] Zhou M. He H. J. Suzuki R. Liu K. X. Tanaka O. Sekiguchi M.. (2007). Localization of sulfonylurea receptor subunits, SUR2A and SUR2B, in rat heart. J. Histochem. Cytochem. 55, 795–804. 10.1369/jhc.6A7104.2007 17438353

